# Exercise preconditioning attenuates cerebral ischemia‐induced neuronal apoptosis, Th17/Treg imbalance, and inflammation in rats by inhibiting the JAK2/STAT3 pathway

**DOI:** 10.1002/brb3.3030

**Published:** 2023-05-04

**Authors:** Yuan Shan, Le Wang, Jingying Sun, Sha Chang, Wei Di, Hua Lv

**Affiliations:** ^1^ Department of Neurology Shaanxi Provincial People's Hospital Xi'an China; ^2^ Central Research Laboratory Shaanxi Provincial People's Hospital Xi'an China

**Keywords:** cerebral ischemia, exercise preconditioning, JAK2/STAT3 pathway, Th17/Treg balance

## Abstract

**Background:**

Exercise preconditioning (EP) is essential for preventing ischemic stroke. Recent studies have shown that EP exerts neuroprotective effects in the cerebral ischemia‐reperfusion injury model. Nonetheless, there have been few reports on the relationship between EP and the Th17/Treg balance. Moreover, it is unclear whether the JAK2/STAT3 pathway is responsible for the neuroprotective effect of EP. Therefore, we aimed to explore the impact of EP, other than the anti‐inflammatory and antiapoptotic functions, on the Th17/Treg balance via the JAK2/STAT3 pathway in a middle cerebral artery occlusion (MCAO)‐induced model.

**Results:**

Fifty rats were randomly allocated into five groups, including the sham group (*n* = 10), EP+sham group (*n* = 10), MCAO group (*n* = 10), EP+MCAO group (*n* = 10), and EP+MCAO+JAK2/STAT3 pathway agonist (coumermycin A1, CA1) group (*n* = 10). The results indicated that EP alleviated neurological deficits, reduced infarct volume, and ameliorated neuronal apoptosis induced by MCAO. Additionally, the MCAO‐induced Th17/Treg imbalance could be rectified by EP. The decreased levels of IL‐10 and Foxp3 and increased IL‐17 and RORα in the MCAO group were reversed by EP treatment. Regarding inflammation, EP reduced the concentrations of IL‐6 and IL‐17 and elevated those of IL‐10 and TGF‑β. The neuroprotective effects of EP were accompanied by decreased phosphorylation of JAK2 and STAT3. Furthermore, CA1 pretreatment diminished all the beneficial effects of EP partially.

**Conclusion:**

Our findings suggest that EP contributes to attenuating neuronal apoptosis, Th17/Treg imbalance, and inflammation induced by MCAO via inhibiting the JAK2/STAT3 pathway, indicating its therapeutic potential in ischemic stroke.

## Graphical abstract

EP alleviates MCAO‐induced neurological deficits.EP attenuates MCAO‐induced neuronal apoptosis, Th17/Treg imbalance, and inflammation in MCAO‐induced rats.EP inhibits abnormal activation of the JAK2/STAT3 signaling pathway in MCAO‐induced rats.

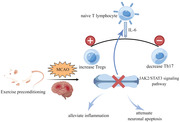

## 1 INTRODUCTION

According to the Global Burden of Disease Study 2019, stroke remains the third‐leading cause of death and disability. Notably, ischemic stroke accounts for 62.4% of all strokes and is accompanied by distinct physical impairment, causing a severe burden globally (Collaborators, 2021). Approximately 3.94 million cases of new stroke and 2.19 million deaths attributed to stroke were reported in China in 2019 (Ma et al., 2021). In a stroke, neurons in the ischemic core quickly undergo necrosis. However, the penumbra surrounding the ischemic center can be rescued if adequate blood perfusion is restored as quickly as possible (Diprose et al., 2021). Currently, endovascular treatment is one of the identified therapies for ischemic stroke in addition to intravenous thrombolysis employing recombinant tissue plasminogen activator (rt‐PA), which is the only US FDA‐approved medicine for ischemic stroke in the ultra‐early stage (Goyal et al., 2016). However, no more than 50% of patients with acute ischemic stroke benefit from either treatment. Moreover, a secondary injury may be further exacerbated after the reperfusion, including hyperperfusion syndrome and even death, known as cerebral ischemia‐reperfusion injury (CIRI) (Huang et al., 2020). Thus, there is an urgent need to uncover the underlying mechanisms of CIRI and seek novel, more reliable strategies for treating ischemic stroke.

Neurological impairment after cerebral ischemia is attributed to various pathophysiological events, including neuronal apoptosis, inflammation, immune dysfunction, and blood‐brain barrier damage (Khoshnam et al., 2017). Apoptosis is a form of programmed cell death induced by cerebral ischemia and controlled by the dynamic balance between pro‐ and antiapoptotic genes (Uzdensky, 2019). As previously reported, the JAK2/STAT3 signaling pathway can mediate apoptosis‐related gene expression, and the upregulation of antiapoptotic genes is beneficial for neuronal apoptosis (Kim et al., 2017; Tang et al., 2020). Additionally, the immune system participates in the pathophysiological course of ischemic stroke (Rayasam et al., 2018). The inflammatory cascade after stroke can result in variations in the immune system. T helper 17 (Th17) cells and CD4^+^CD25^+^ regulatory T cells (Tregs) are two types of CD4+ T lymphocytes with antagonistic roles and maintain the relative immune balance (Lee, 2018). Interleukin‐17 (IL‐17) is an important cytokine involved in inflammation‐related diseases and is mainly secreted by Th17 cells, which express retinoic acid‐related orphan receptor α (RORα) and promote interleukin‐6 (IL‐6) production. Tregs can infiltrate the ischemic brain and protect against stroke‐associated damage. Tregs mainly secrete anti‐inflammatory cytokines, such as interleukin‐10 (IL‑10) and transforming growth factor beta (TGF‑β), specifically express transcription factor forkhead box P3 (Foxp3) and play an essential role in immune tolerance (Bellone et al., 2020; Goschl et al., 2019). Maintaining the Th17/Treg balance is crucial for inflammatory and autoimmune diseases. One previous study demonstrated that the tyrosine kinase inhibitor tyrphostin AG126 could alleviate the inflammatory response by increasing Foxp3^+^ regulatory T cells and reducing IL‐17 levels in a mouse model of adjuvant‐induced arthritis (Ahmad et al., 2016). Furthermore, a study related to acute lung injury revealed that inhibition of IL‐2‐inducible T‐cell kinase signaling caused a reduction in airway inflammation by regulating the Th17/Treg immune balance (Nadeem et al., 2020). These studies shed light on the assumption of our present study. To the best of our knowledge, limited studies have examined the Th17/Treg immune balance in stroke. After a stroke, peripheral leukocytes are recruited and infiltrate the ischemic region, amplifying inflammatory reactions and inducing neuronal apoptosis (Iadecola et al., 2020). IL‐6 is a cytokine engaged in the proinflammatory process after stroke, which is mediated by the Janus kinase 2 (JAK2) and the signal transducer and activator of transcription 3 (STAT3) pathway (Hunter & Jones, 2015). This pathway has been reported to be regulated by remote ischemic preconditioning in myocardial ischemia‐reperfusion injury research (Sawashita et al., 2020). Additionally, a study on autism found that resveratrol could compete with neuroimmune dysfunction by downregulating proinflammatory cytokines, such as IL‐6, and the JAK1/STAT3 pathway in BTBR mice (Ahmad, Ansari, et al., 2018). Moreover, inhibition of the JAK2/STAT3 pathway is essential in neuroprotection after CIRI (Fan & Zhou, 2021). The JAK2/STAT3 pathway plays a critical role in the inflammatory reaction, which is necessary for the function of cytokines, the differentiation of T lymphocytes, and the activation of adaptive immunity (O'Shea et al., 2015). Interestingly, the JAK2/STAT3 pathway exhibits bidirectional effects in the related mechanisms of CIRI (Zhong et al., 2021). Moreover, the differentiation of naive T lymphocytes into Th17 cells or Tregs partly relies on IL‐6, and the activation of STAT‐3 may contribute to the induction of Th17 differentiation (Lin et al., 2017). Therefore, we speculate on the possible links between the Th17/Treg balance, inflammation, neuronal apoptosis, and the JAK2/STAT3 signaling pathway in the mechanisms of CIRI.

Exercise preconditioning (EP) might display endogenous neuroprotective effects by inducing ischemic tolerance (Alkadhi, 2018). EP is safe, convenient, and feasible compared to other preconditioning methods, such as ischemic preconditioning (Zhao et al., 2019). The findings of both clinical and preclinical studies have indicated that physical activity positively correlates with neurological function (Kramer et al., 2019; Shamsaei et al., 2017). EP before cerebral ischemia has notably improved neurological deficits, inhibited neuronal apoptosis, increased cerebral blood flow, and promoted angiogenesis (Correia et al., 2021; Liu et al., 2021). One previous study found that EP elicited neuroprotective effects by alleviating inflammation in the ischemic brain via regulating the TLR4/NF‐κB signaling pathway (Zhu et al., 2016). Another study suggested that EP for at least 5 days per week for 3 weeks could reduce the infarct size and improve neurological function after focal ischemia. Therefore, the impact of exercise preconditioning depends on the specific intensity, frequency, and duration.

To the best of our knowledge, limited studies have focused on the JAK2/STAT3 signaling pathway and Th17/Treg imbalance in the underlying mechanisms of EP on CIRI. Therefore, we aimed to determine the potential impact of EP on middle cerebral artery occlusion (MCAO)‐induced neuronal apoptosis, Th17/Treg imbalance, and inflammation in rats. Furthermore, to explore the underlying mechanism, a JAK2 agonist (Coumermycin A1) was employed to activate the JAK2/STAT3 pathway, and explore its potential role.

## 2 MATERIALS AND METHODS

### 2.1 Experimental animals

Adult male Sprague‐Dawley rats (8 weeks old, weighing 250–280 g) were purchased from Vital River Laboratory Animal Technology Co., Ltd. (Beijing, China). For 3 days before the experiment, the rats were housed under controlled room temperature (approximately 22–25°C), with a relative humidity of approximately 50%, and a 12 h light‐12 h dark cycle with sufficient access to food and water. All experiments were conducted following the Laboratory Guidelines for the Care and Use of Experimental Animals published by the US National Institutes of Health. All of the protocols were reviewed and approved by the Institutional Animal Care and Use Committee of Shaanxi Provincial People's Hospital (No. 2021112).

### 2.2 Exercise preconditioning (EP) protocol

The detailed EP protocol consisted of acclimatized treadmill training for 3 days at 15–20 m/min, 20 min/day, and subsequently formal treadmill training for 4 weeks, 5 days per week, set at a speed of 25 m/min, 30 min/day. The electric stimulation intensity was 1.5 mA. The entire pretreatment was conducted at room temperature during the daytime.

### 2.3 Establishment of the middle cerebral artery occlusion (MCAO) model

The MCAO model was established after 4 weeks of EP. Briefly, rats were anesthetized with 2% isoflurane. Then the right common carotid artery (CCA), the internal carotid artery (ICA), and the external carotid artery (ECA) were carefully isolated. Next, ligation was conducted proximal to the CCA and ECA. Subsequently, a methylpolysiloxane‐coated nylon monofilament was softly inserted from the CCA and passed by the ICA to the origin of the MCA at approximately 18–20 mm. After 2 h of embolization, the nylon monofilament was finally pulled out. Thus, the cerebral ischemia‐reperfusion injury model was successfully established.

### 2.4 Experimental grouping

Fifty rats were randomly distributed into five groups, including the sham group (*n =* 10), MCAO group (*n =* 10), EP+sham group (*n =* 10), EP+MCAO group (*n =* 10), and EP+MCAO+CA1 group (*n =* 10) according to the random number table method. Coumermycin A1 (CA1), a JAK2 agonist, was employed to activate the JAK2/STAT3 pathway. All of the sham and MCAO group rats were allowed free activity in cages for 4 weeks before surgery. The EP+sham, EP+MCAO, and EP+MCAO+CA1 groups were subjected to a treadmill training protocol. With regard to the surgical procedures, the sham and EP+sham groups were subjected to all steps except for the arterial occlusion. Thirty minutes before reperfusion, CA1 (100 μg/kg) was administered intraperitoneal injection in the EP+MCAO+CA1 group, as reported previously (Gong et al., 2018). By contrast, equal normal saline was given to the other groups. During the operation, the animal's body temperature was maintained at approximately 37°C using a heating blanket.

### 2.5 Assessment of neurological function

An investigator blinded to the procedures evaluated the rats’ neurological functions according to the Longa score 24 h and 72 h after reperfusion (*n =* 8‐10/group). The specific criteria were as follows (5‐point scale): grade 0, no neurological defect; grade 1, failure of affected lateral forepaw extension, indicating a mild neurological deficit; grade 2, turning around to the paralyzed side while walking, suggesting a moderate neurological deficit; grade 3, susceptible to falling on the paralyzed side, demonstrating a severe neurological deficit; and grade 4, inability to walk spontaneously and unconsciousness. A neurological deficit score between 1 and 3 was considered a successful model.

### 2.6 TTC (2,3,5‐triphenyl tetrazolium chloride) staining

Rats were sacrificed after anesthesia with 5% isoflurane, and then brain tissues were collected and transferred to ‐80°C after being frozen in liquid nitrogen (*n =* 5/group). The ischemic brain regions were sliced into 2‐mm‐thick consecutive segments from the frontal tip and stained with 2% 2,3,5‐triphenyl tetrazolium chloride (TTC) solution (Sigma‐Aldrich, St. Louis, MO, USA) in phosphate‐buffered saline (PBS) at 37°C for 15–30 min. After 4% paraformaldehyde fixing and staining, the dyeing film was imaged, and the infarct volume was analyzed using Image J software (National Institutes of Health, Bethesda, MA, USA).

### 2.7 Terminal deoxynucleotidyl transferase dUTP nick‐end labeling (TUNEL) staining

After reperfusion for 72 h, the rats’ ischemic brain tissues, including the hippocampal tissue region, were cut into 4–8 μm‐thick slices and stained following the In Situ Cell Death Detection Kit instructions provided by the manufacturer (Sigma‐Aldrich). After staining, we randomly selected five visual fields to calculate positive cells using a fluorescence microscope. The ratio of TUNEL‐positive cells to the total cells was defined as the apoptosis rate and was calculated by an investigator blinded to the experiment using Image J software (National Institutes of Health, Bethesda, MA, USA).

### 2.8 Nissl's staining

Seventy‐two hours following reperfusion, the rats’ brain tissues were prepared to estimate the neuronal damage by applying Nissl's staining. Briefly, 5‐μm‐thick slices of paraffin sections were routinely dewaxed to water with xylene I and xylene II for 15 min, respectively, before implementing gradient alcohol dehydration (100%, 100%, 95%, 90%, 80%, 70%, and 50% each for 5 min). After washing with distilled water, the brain tissues were dyed in Nissl staining solution (Cat. No. G3661; Beijing Solarbio Technology Co., LTD.) for 40 min in a 60°C incubator. After washing with distilled water, the brain sections were dehydrated with gradient ethanol, cleared with xylene, and finally sealed with neutral gum. The images were observed at ×200 magnification using a DXS‐3 inverted biological microscope (Shanghai Dilun Optical Instrument Co., LTD). The results were analyzed by Image J software.

### 2.9 Flow cytometry

To investigate the Th17 and Treg cells, peripheral blood mononuclear cells were first separated and prepared. The percentages of Th17 and Treg cells and the Th17/Treg ratio were ascertained by flow cytometry as previously described (Zhang et al., 2021). The antibodies used in the experiment, including APC‐Cy7‐conjugated CD3, fluorescein isothiocyanate (FITC)‐conjugated CD4, APC‐conjugated CD25, PE‐conjugated Foxp3, and PE‐conjugated IL‐17, were obtained from eBioscience (San Diego, CA, USA). Data were determined by a flow cytometer (Beckman Coulter, Fullerton, CA, USA).

### 2.10 Quantitative real‐time PCR (qRT‐PCR)

According to the manufacturer's instructions, total RNA was extracted from brain cells by applying TRI Reagent Solution (Ambion, Carsland, CA, USA). Subsequently, cDNA was synthesized using a reverse transcription kit (Promega, Madison, WI, USA). qRT‐PCR was employed to verify the relative expression of IL‐10, Foxp3, IL‐17, and RORα mRNA. The relative mRNA levels of target genes were normalized to β‐actin identified as an internal reference, using the 2^–∆∆Ct^ method (Schmittgen & Livak, 2008). All of the procedures were repeated three times. The specific primers were as follows: IL‐10, 5’‐AGTGGAGCAGGTGAAGAATG‐3’ and 5’‐GAGTGTCACGTAGGCTTCTATG‐3’; Foxp3, 5’‐ACTGGAGTCTTCTCCCTCAA‐3’ and 5’‐TGGGAAGGTGCAGAGTAGA‐3’; IL‐17, 5’‐AAACGCCGAGGCCAATAA‐3’ and 5’‐GAAGTGGAACGGTTGAGGTAG‐3’; RORα, 5’‐GCACCTCCAGCCATTTATCT‐3’ and 5’‐ATGAGGTTTCGTCACAGTATGG‐3’; β‐actin, 5’‐AGATGTGGATCAGCAAGCAG‐3’ and 5’‐GCGCAAGTTAGGTTTTGTCA‐3’.

### 2.11 Enzyme‐linked immunosorbent assay (ELISA)

The concentrations of IL‐6, IL‐17, IL‐10, and TGF‐β in serum were measured with ELISA kits (Thermo Fisher Scientific), following the manufacturer's instructions. All samples were tested in triplicate for statistical analysis.

### 2.12 Western blot

Western blot was employed to detect the specific protein expression of JAK2, p‐JAK2(Tyr1007/1008), STAT3, p‐STAT3(Tyr705), Foxp3, RORα, Bcl‐2, and cleaved caspase‐3. The ischemic brains were ordered to prepare tissue homogenate to obtain the total protein using a protein extraction kit (Beyotime, Shanghai, China), following the manufacturer's protocol. In brief, equal amounts of extracted proteins were taken for standardized SDS‐PAGE electrophoresis. The proteins were transferred onto the polyvinylidene fluoride (PVDF) membranes (Bio‐Rad, Hercules, CA, USA), which were sequentially incubated with 5% skim milk at room temperature for approximately 1 h. Subsequently, the membranes were incubated with corresponding primary antibodies and then added to the TBST buffer to incubate overnight at 4°C. The following primary antibodies were used: anti‐JAK2 (ab108596; 1:5000 dilution; Abcam), anti‐pJAK2 (ab32101; 1:5000 dilution; Abcam), anti‐STAT3 (ab31370; 1:500 dilution; Abcam), anti‐pSTAT3 (ab76315; 1:2000 dilution; Abcam), anti‐Foxp3 (ab215206; 1:1000 dilution; Abcam), anti‐RORα (ab256799; 1:500 dilution; Abcam), anti‐Bcl2 (ab196495; 1:1000 dilution; Abcam), and anti‐cleaved caspase‐3 (ab13847; 1:500 dilution; Abcam). GAPDH (ab181602; 1:5000 dilution; Abcam) and β‐actin (ab8227; 1:5000 dilution; Abcam) were used as internal references. Finally, the goat antirabbit IgG conjugated by HRP secondary antibody (ab205718; 1:20,000 dilution; Abcam) was added and incubated for 1 h at room temperature. Then the membranes were incubated with an ECL kit (Thermo Fisher Scientific, San Jose, CA, USA) to develop protein signals. Semiquantitative analysis was accomplished using β‐actin and DAPDH as the internal references. The experiments were repeated in triplicate.

### 2.13 Statistical analysis

Statistical analysis, including parametric and nonparametric tests, was performed using SPSS 26.0 (IBM Corp., Armonk, NY, USA) and GraphPad Prism 8.0.1 (GraphPad Software, Inc., La Jolla, CA, USA). Normally distributed data are shown as the mean ± standard deviation (SD) and determined using one‐way analysis of variance (ANOVA) followed by Bonferroni's post hoc analysis for comparisons among groups if the equal variance was supposed. The Kruskal‐Wallis test was applied to accomplish nonparametric analyses. The Shapiro‐Wilk test was used to test the normal distribution of all data. *p* Values < .05 were considered to indicate a statistically significant difference.

## 3 RESULTS

### 3.1 EP alleviated neurological deficits and reduced infarct size

**FIGURE 1:**
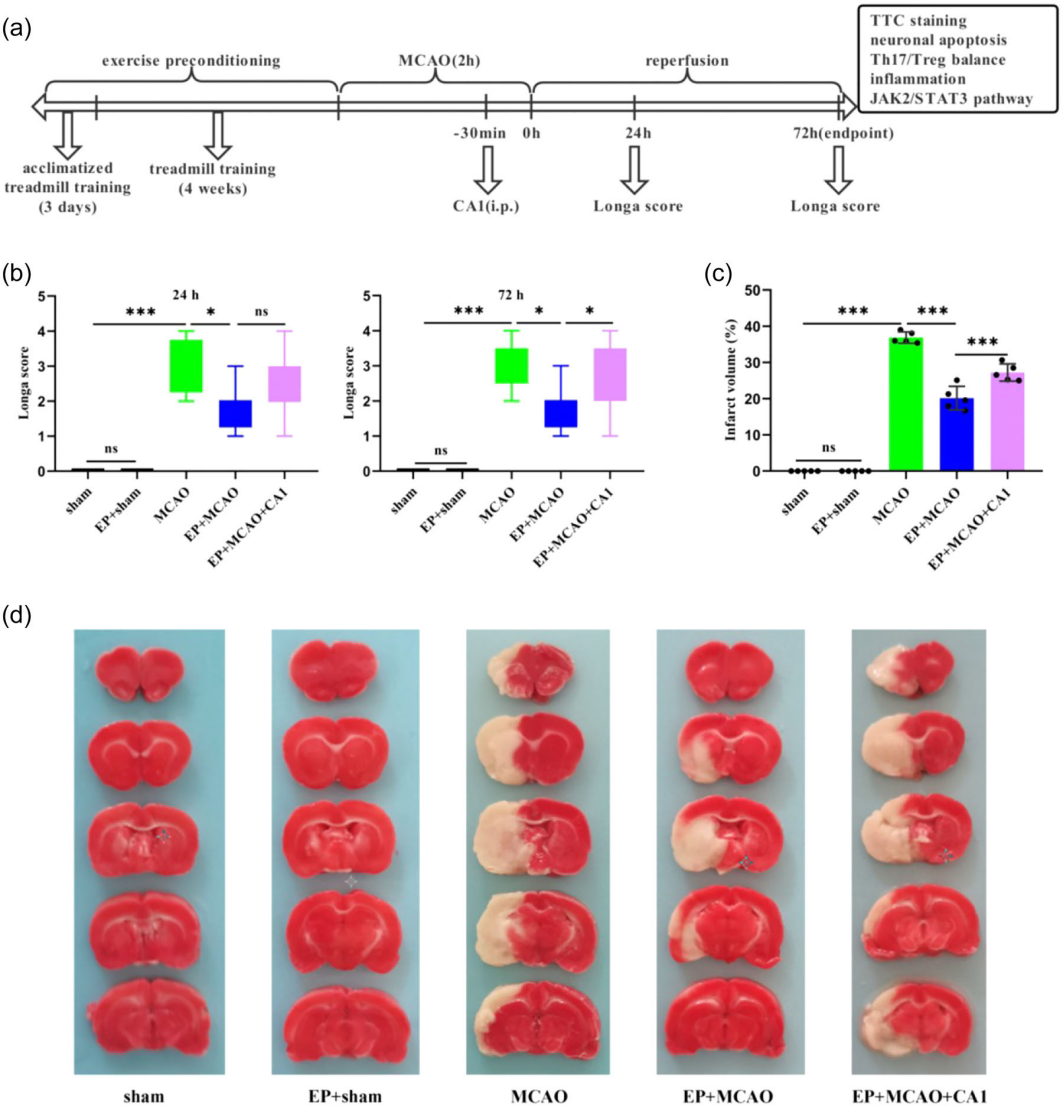
EP alleviated MCAO‐induced cerebral injury and neurological deficits. (a) Flow diagram of the intact experimental protocol. (b) The neurological deficit score was measured 24 h and 72 h after reperfusion (*n =* 8‐10/group). (c) Graphical representation of the infarct volume ($\bar{x}$ ± s, *n =* 5/group). (d) The infarction tissues were characterized and detected by TTC staining 72 h after reperfusion. *p < .05; *** *p* < .001; ns: not significant.

**FIGURE 2:**
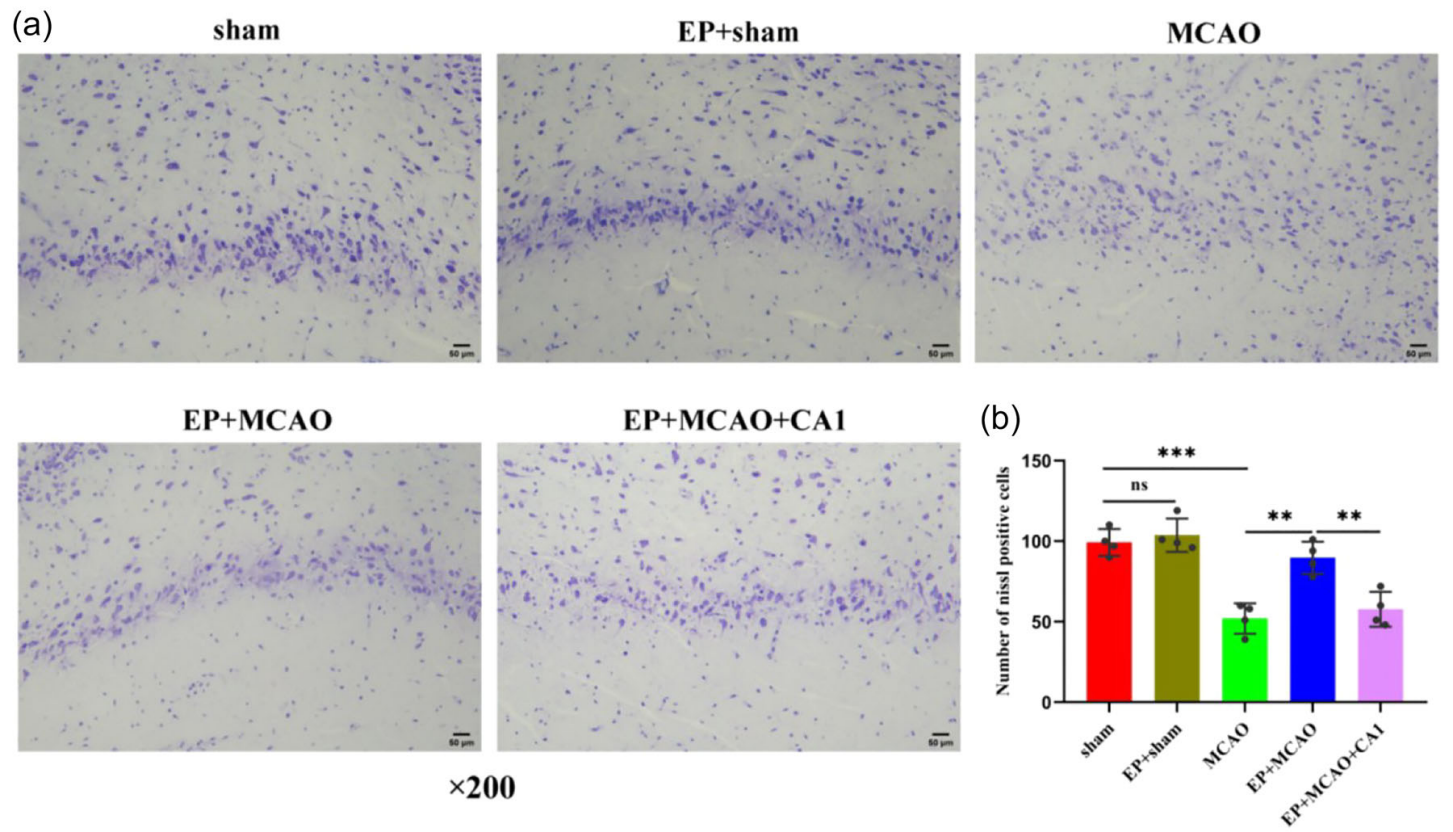
EP ameliorated MCAO‐induced neuronal loss ($\bar{x}$ ± s, *n =* 4/group). (a) Seventy‐two hours following reperfusion, the neuronal loss observed in rat brain tissues was determined by Nissl's staining (magnification ×200, scale bar = 50 μm). (b) Graphical representation of the Nissl‐positive cells in hippocampus CA1. **p < .01; ***p < .001; ns: not significant.

The flow chart of the experiment is shown in Figure 1a. As shown in Figure 1b, compared to the sham and EP+sham groups, the MCAO group demonstrated worse neurological function (*p* < .001), while EP significantly ameliorated the neurological deficits (*p* < .05). Moreover, CA1 pretreatment reversed the protective effect of EP, especially after 72 h reperfusion (*p* < .05). The infarct volume was significantly increased in the MCAO group compared to the sham and EP+sham groups (*p* < .001) but decreased substantially as a result of EP (*p* < .001). In addition, after CA1 pretreatment, the infarct size was amplified again compared to the EP+MCAO group (Figure 1c and d). Furthermore, Nissl staining results showed that EP could mitigate MCAO‐induced neuron damage (Figure 2a and b). These results indicated that EP could protect against ischemic brain damage in rats. CA1 pretreatment might offset the protective effect of EP.

### 3.2 EP inhibited neuronal apoptosis in MCAO rats

**FIGURE 3:**
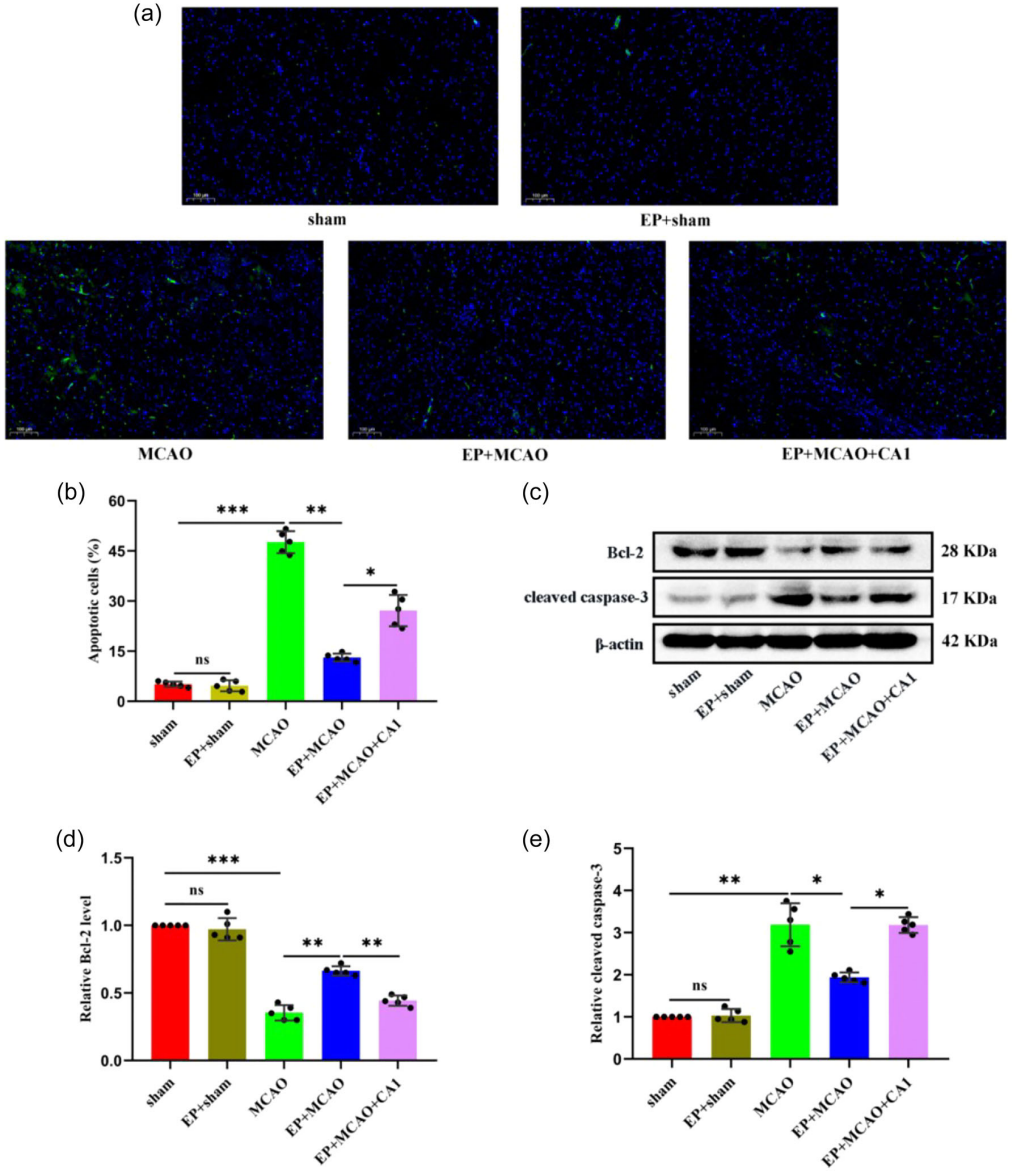
EP attenuated MCAO‐induced apoptosis ($\bar{x}$ ± s, *n =* 5/group). (a) Neuronal apoptosis was detected by TUNEL staining (scale bar = 100 μm). (b) Graphical representation of the apoptotic cell ratio. (c‐e) The relative expression of apoptosis‐related genes, including Bcl‐2 and cleaved caspase‐3, was determined by western blot. *p < .05; **p < 0.01; ***p < .001; ns: not significant.

Next, TUNEL assay and western blot were employed to explore neuronal apoptosis. The apoptotic cell ratio was significantly higher in the MCAO group than in the sham and EP+sham groups (*p* < .001), while EP markedly alleviated neuronal apoptosis (*p* < .01). In addition, neuronal apoptosis was aggravated after CA1 pretreatment compared with the EP+MCAO group (Figure 3a and b).

Next, the relative protein expression of apoptosis‐related genes was determined to verify the role of EP. As presented in Figure 3c‐e, we found significantly upregulated expression of caspase‐3 and downregulated expression of Bcl‐2 in the MCAO group compared to the sham and EP+sham groups (*p* < .01 and *p* < .001, respectively), which could be reversed by EP (*p* < .05 and *p* < .01, respectively). However, CA1 pretreatment impaired the antiapoptotic effect of EP (*p* < .01 and *p* < .05). These findings suggest that EP is critical in inhibiting neuronal apoptosis via the JAK2/STAT3 signaling pathway.

### 3.3 EP modulated the dynamic balance between Th17 and Treg cells

**FIGURE 4:**
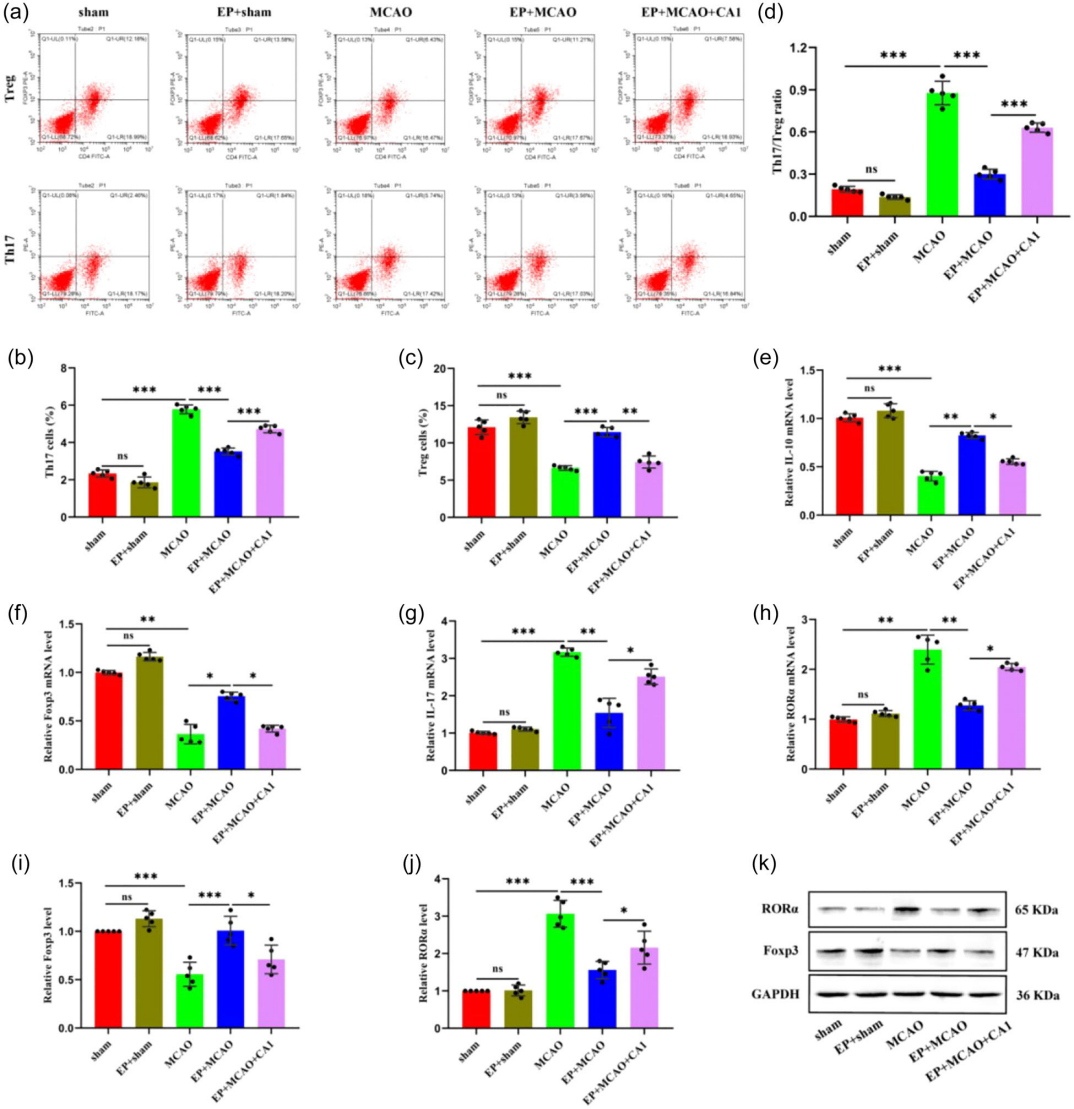
EP rectified the MCAO‐induced Th17/Treg imbalance. (a) The changes in Th17 and Treg cells were analyzed by flow cytometry. (b, c) The proportions of Th17 and Treg cells and (d) the Th17/Treg ratio were determined and analyzed ($\bar{x}$ ± s, *n =* 5/group). The relative mRNA levels of IL‐10 (e), Foxp3 (f), IL‐17 (g), and RORα (h) were estimated by qRT‐PCR ($\bar{x}$ ± s, *n =* 5/group). Western blot was applied to assess the protein expression of Foxp3 (i, k) and RORα (j, k). *p < .05; **p < .01; ***p < .001; ns: not significant.

As shown in Figure 4a, the flow cytometry results suggested cerebral ischemia might induce changes in Th17 and Treg cells. Notably, the percentage of Th17 cells was elevated in the MCAO group compared to those in the sham and EP+sham groups, consistent with the Th17/Treg ratio (Figure 4b and d). However, the Treg cells showed a negative trend (Figure 4c). It was interesting to note that EP may positively ameliorate the Th17/Treg imbalance. Furthermore, CA1 pretreatment weakened the beneficial impact of EP (*p* < .01 or *p* < .001).

qRT‐PCR was performed to estimate the mRNA levels of IL‐10, IL‐17, FoxP3, and RORα. We observed that the mRNA levels of IL‐10 and Foxp3 were lower in the MCAO group than in the sham and EP+sham groups (Figure 4e and f). Conversely, the IL‐17 and RORα levels were notably higher in the MCAO group (Figure 4g and h). EP could reverse the above results, but after CA1 pretreatment, IL‐17 and RORα increased, and IL‐10 and Foxp3 decreased compared to those in the EP+MCAO group. Moreover, the protein expression of Foxp3 and RORα was assessed by western blot. The results revealed that the decrease in Foxp3 and increase in RORα in the MCAO group were reversed by EP but diminished by CA1 (Figure 4i–k). These results demonstrate that EP contributes to the dynamic balance between Th17 and Tregs by inhibiting the activation of the JAK2/STAT3 pathway.

### 3.4 EP influenced the levels of inflammatory cytokine associated with Th17 and Tregs

**FIGURE 5:**
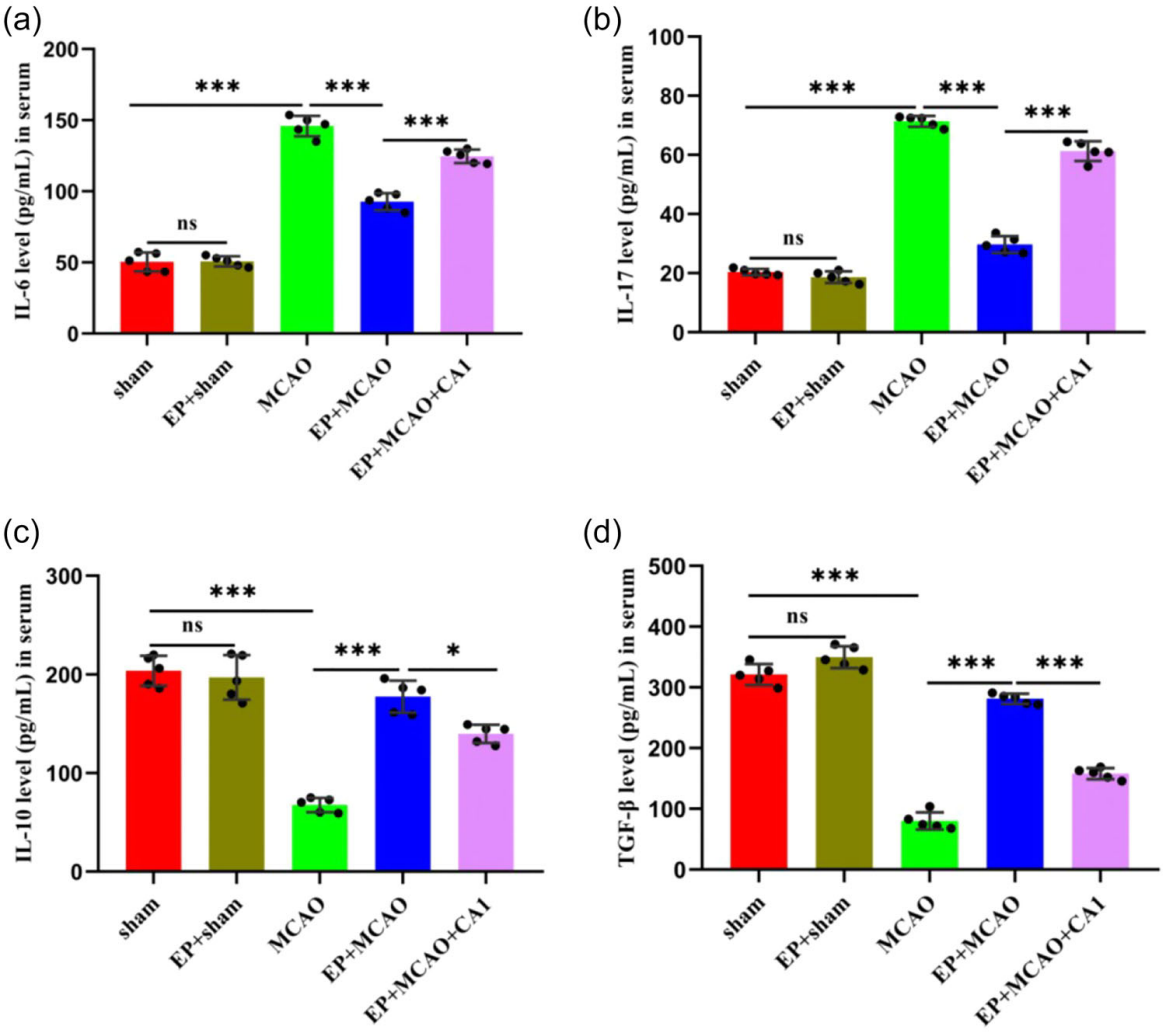
EP influenced the inflammatory cytokine levels associated with Th17 and Tregs. ELISA was performed to measure the levels of IL‐6 (a), IL‐17 (b), IL‐10 (c), and TGF‐β (d) in serum ($\bar{x}$ ± s, *n =* 5/group). *p < .05; ***p < .001; ns: not significant.

ELISA was adopted to determine the levels of IL‐6, IL‐17, IL‐10, and TGF‐β. The results demonstrated that EP significantly reduced the elevation of IL‐6 and IL‐17 in the EP+MCAO group compared to the MCAO group (all *p* < .001) (Figure 5a and b). In contrast, the IL‐10 and TGF‐β levels increased substantially in the EP+MCAO group (all *p* < .001) (Figure 5c and d). Interestingly, CA1 pretreatment could partially offset the impact of EP (*p* < .001, *p* < .05). These results suggest that EP relieves the immune‐inflammatory process associated with Th17 and Treg cells, while CA1 pretreatment weakens the anti‐inflammatory effect of EP.

### 3.5 EP attenuated the abnormal activation of the JAK2/STAT3 signaling pathway

**FIGURE 6:**
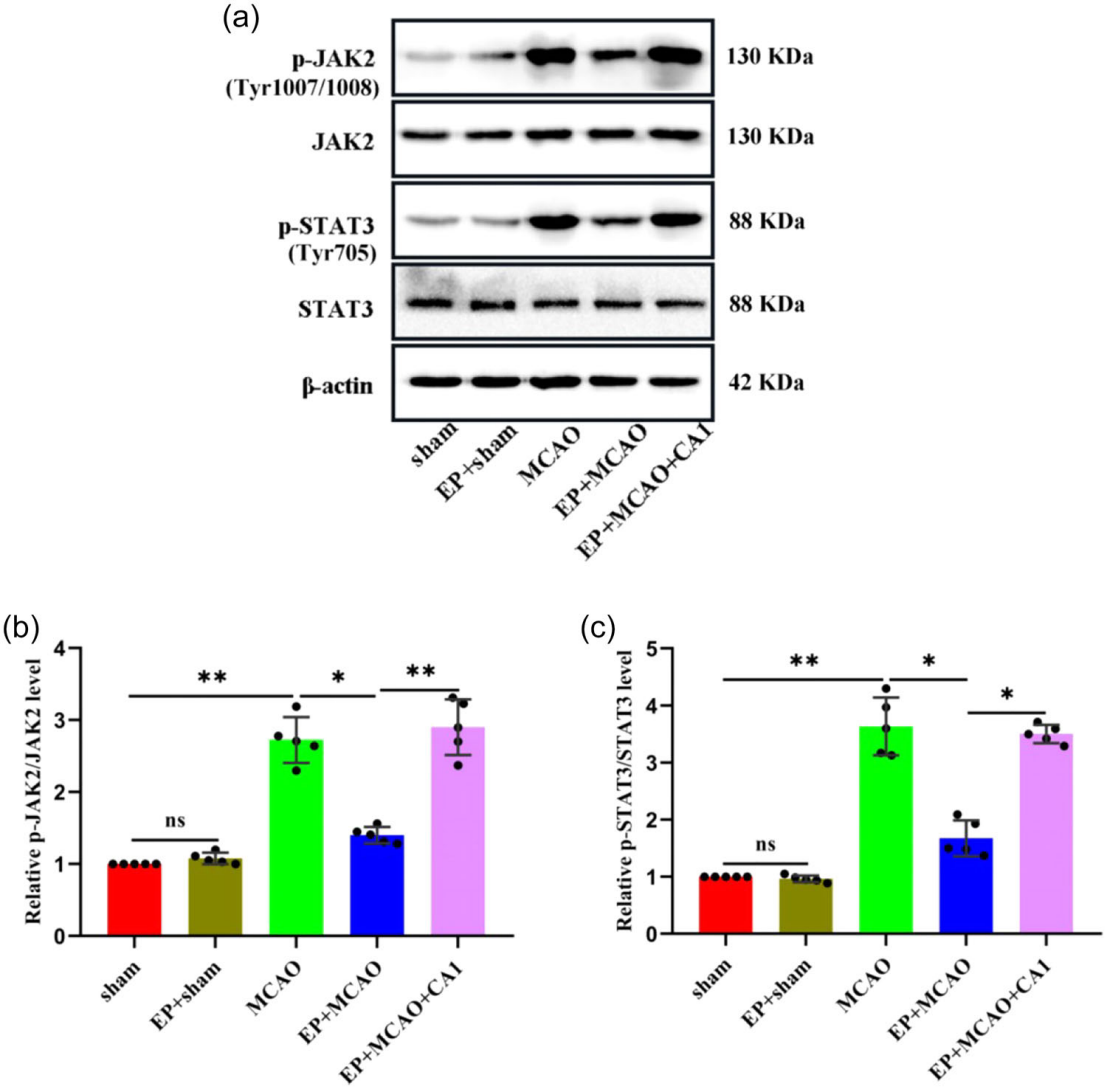
EP inhibited the abnormal activation of the JAK2/STAT3 signaling pathway. (a) Western blot was employed to determine the protein expression of JAK2, p‐JAK2, STAT3, and p‐STAT3. (b, c) Graphical representation of p‐JAK2 and p‐STAT3 normalized to JAK2 and STAT3, respectively ($\bar{x}$ ± s, *n =* 5/group). *p < .05; **p < .01; ns: not significant.

Western blot was applied to determine the expression of genes associated with the JAK2/STAT3 pathway (Figure 6a). Compared with the sham and EP+sham groups, the phosphorylation of JAK2 and STAT3 was higher in the MCAO group (all *p* < .01). However, EP significantly downregulated pJAK2(Tyr1007/1008) and pSTAT3(Tyr705) expression (all *p* < .05), and CA1 pretreatment reversed the trend of EP (Figure 6b and c). These results confirm that EP inhibits the abnormal activation of JAK2 and STAT3, playing a pivotal role in protecting against CIRI.

## 4 DISCUSSION

**FIGURE 7:**
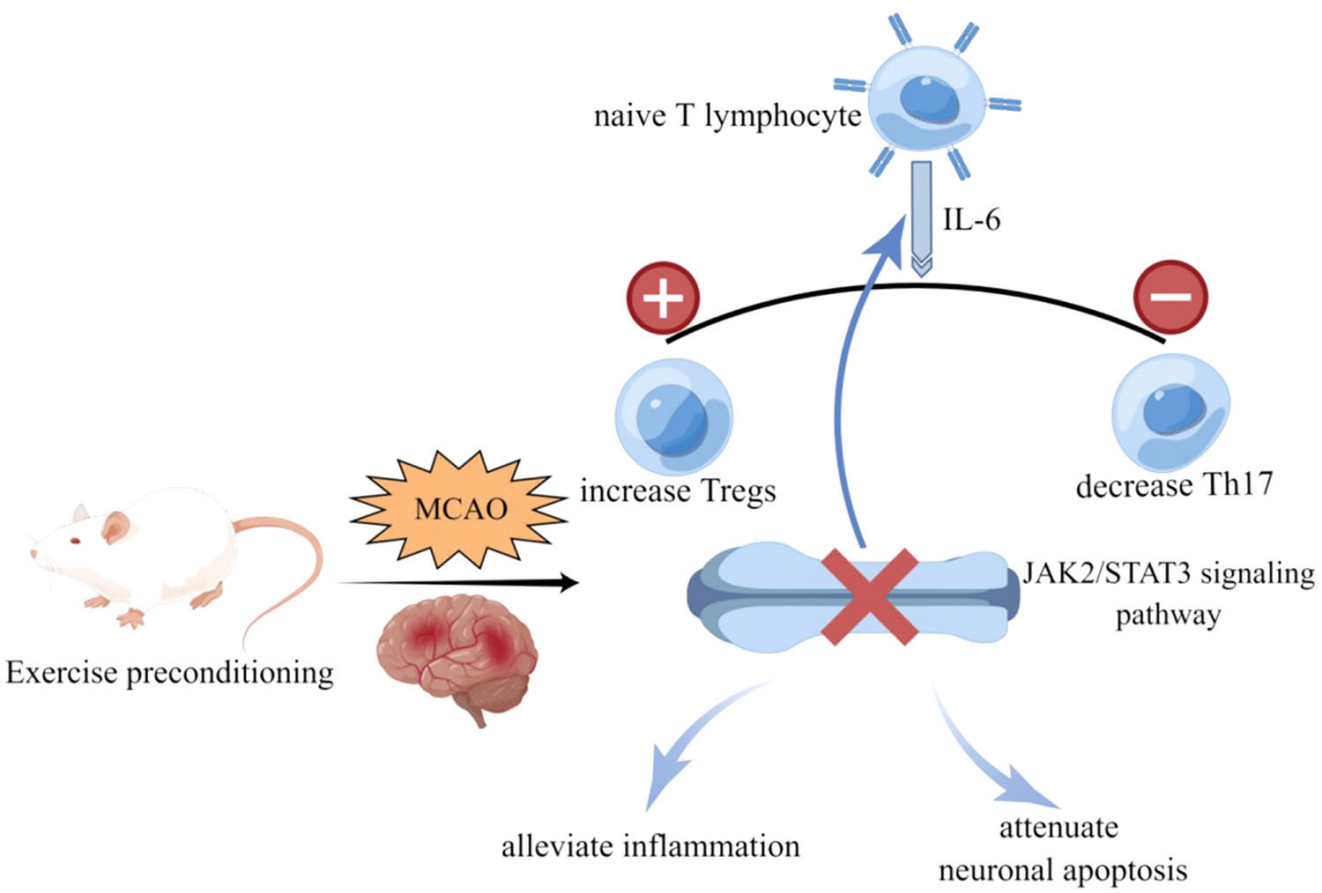
A graphic representation of the underlying mechanism of exercise preconditioning.

Although previous studies have revealed that pre‐stroke physical activity can improve functional prognosis after cerebral ischemia and reduce the risk of admission severity and mortality, the underlying mechanisms have not been thoroughly explained (Abbott et al., 1994; Hung et al., 2021; Lee et al., 2003). In this study, we provide evidence of the neuroprotective effect of EP, which is mediated by the alleviation of neuronal apoptosis, inflammation, and Th17/Treg imbalance in the MCAO‐induced ischemia‐reperfusion injury model. EP was also found to attenuate the abnormal activation of the JAK2/STAT3 signaling pathway after cerebral ischemia, indicating the potential significance of the JAK2/STAT3 pathway in the neuroprotective effects of EP (Figure 7).

EP is similar to ischemic preconditioning, demonstrating endogenous neuroprotective effects after stroke. The most common modes of EP include voluntary wheel running activity and forced treadmill running, both of which have neuroprotective effects (Kalogeraki et al., 2016; Otsuka et al., 2016). Moderate‐intensity exercise includes treadmill running at 15–25 m per min, while a speed surpassing 25 m/min is defined as high‐intensity exercise. It is well‐established that both moderate‐ and high‐intensity exercises have neuroprotective effects (Hafez et al., 2020; Otsuka et al., 2019). One study indicated that EP at least thrice a week reduced the risk of ischemic brain injury (Terashi et al., 2019). Based on the above research, we affirmed that exercise intensity and frequency before ischemia are essential to stroke outcomes, and accordingly, we adopted the mature treadmill training protocol. In line with previous studies, our results revealed that EP could significantly reduce infarct volume and neuronal damage, accompanied by a notable improvement in neurological deficits.

Apoptosis of neuronal cells is essential to the pathophysiology of ischemic stroke, especially in the penumbra region. Many apoptosis‐related genes are involved, among which the core members are HSP, Bcl‐2, Bax, and caspase‐3. Previous study have demonstrated that treadmill training could alleviate neurological deficits by protecting HSP72‐containing neurons in rats (Wang et al., 2019). In addition, activation of the ERK1/2 signaling pathway might attenuate apoptosis due to CIRI by regulating Bcl‐2 and Bax expression (Li et al., 2021). Nevertheless, the impact of EP on cerebral ischemia‐induced hippocampal impairment remains controversial. Studies have concluded that pre‐ischemic exercise exhibited antiapoptosis effects on hippocampal CA1 and CA3 neurons by modulating apoptosis‐related genes (Aboutaleb et al., 2015; Shamsaei et al., 2015). Accordingly, we explored neuronal apoptosis and the expression of Bcl‐2 and cleaved caspase‐3 by TUNEL assay and western blot, respectively. Moreover, Nissl staining was applied to determine neuronal damage in the hippocampal regions of the rats. As expected, we observed a reduction in the apoptotic and necrotic cells, upregulated expression of Bcl‐2, and downregulated expression of cleaved caspase‐3 in the EP+MCAO group compared to the MCAO group, indicating that EP prior to cerebral ischemia exhibited an antiapoptotic effect.

Numerous studies have focused on the mechanism related to Th17 and Tregs, given that their imbalance is thought to be responsible for stroke‐related brain injury. In addition, the appropriate expression of cytokines secreted by Th17 and Tregs, such as IL‐6, IL‐10, TGF‐β, and IL‐17, contribute to maintaining the Th17/Treg balance (Goodman et al., 2012). Previous reports have shown that oxidized low‐density lipoprotein leads to Th17/Treg imbalance in patients with atherosclerotic cerebral infarction (Li et al., 2014). It has been reported that EP combined with 17‐estradiol could reduce MMP9 and increase IL‐10 levels, playing a protective role in an ovariectomized mouse model (Naderi et al., 2018). Treg cells are crucial in neuronal protection against stroke and recovery (Ito et al., 2019; Shi et al., 2021). Furthermore, a previous study highlighted that IL‐33 could ameliorate cerebral ischemia in the MCAO model by suppressing the Th17 response (Luo et al., 2015). Interestingly, no previous reports have explored whether EP is associated with maintaining the Th17/Treg balance. In the present study, we focused on detecting the amount of Th17 and Treg cells and the expression of their specific transcription factors. Consistent with previous studies, our results revealed that EP could reduce the Th17/Treg ratio and Th17 cell proportion and elevate the Treg cells, indicating that EP might be critical in regulating the MCAO‐induced Th17/Treg imbalance.

Interrupting cerebral blood flow in conjunction with reperfusion after ischemia can induce inflammation‐related cytokines and mediators, such as interleukins, to infiltrate the brain and promote follow‐up inflammatory responses (Zhang et al., 2021). Microglia, known as the brain tissue macrophages, play a pivotal role by producing IL‐6 in response to ischemia (Mizuma & Yenari, 2017). Studies have shown that EP can reduce proinflammatory cytokines, such as TNF‐α and IL‐6, thereby inhibiting inflammation in ischemic stroke (Ding et al., 2005; Radak et al., 2016). One study revealed that the neuroprotection in cerebral ischemia was attributed to the exercise‐induced hormone irisin via activation of the Akt and ERK1/2 signaling pathway (Li et al., 2017). Moreover, treadmill training could relieve cerebral ischemia‐reperfusion injury by facilitating M2 microglia activation through IL‐4 upregulation mediated by the JAK1/STAT6 pathway (Lu et al., 2021). Therefore, there is a need to seek therapies targeting the inflammatory response. In the present study, we mainly focused on investigating the levels of inflammation‐related cytokines secreted by Th17 and Treg cells. Similar to previous research, our results showed that IL‐6 and IL‐17 levels increased significantly in the MCAO group compared to the sham and EP+sham groups but that the IL‐10 and TGF‐β levels were lower. Additionally, EP reversed the trends, suggesting that EP mitigates MCAO‐induced immune inflammation by modulating the inflammatory cytokines.

Increasing studies have demonstrated that the JAK2/STAT3 signaling pathway is activated immediately after cerebral ischemia, plays a pivotal role in microglia activation, and acts as a potential therapeutic hotspot in neuroinflammation and neuronal apoptosis (Amani et al., 2019; Wu et al., 2018). It has been reported that activation of the JAK2/STAT3 pathway could induce the production of IL‐6 and exacerbate stroke‐induced brain injury (Schindler et al., 2007). Interestingly, the mechanisms involved in stroke through IL‐6 and the JAK2/STAT3 pathway may be more complex, likely accompanied by immune system dysfunction, which is of great importance not only in the early stage but also in the later period ischemic stroke (Schmidt‐Pogoda et al., 2019). It is well‐known that IL‐6 and IL‐10 are upstream mediators of STAT3, while STAT3 is a transcriptional regulator of IL6, as verified previously by dual luciferase reporter assay (Wu et al., 2022). Remarkably, the differentiation of naive T lymphocytes into Th17 or Tregs partly depends on IL‐6, and the activation of STAT‐3 may contribute to the induction of Th17 cell differentiation. Previous studies by Sheikh F. Ahmad et al. identified the adenosine A2A receptor agonist and PPARδ agonist GW0742 as potential therapeutic targets for autism by decreasing the levels of IL‐17A, RORγt, Stat3, and pStat3 and increasing the protein and mRNA expression of Foxp3 and IL‐10 (Ahmad et al., 2018; Ansari et al., 2017). To illustrate the underlying mechanism of EP, we raised a presumption that EP might exert neuroprotective effects in the CIRI model by affecting neuronal apoptosis, inflammation, and the Th17/Treg balance via regulating the JAK2/STAT3 pathway. Indeed, coumermycin A1 (CA1), a JAK2 agonist, has been shown to induce JAK2 phosphorylation and activate the JAK2/STAT3 pathway (Gong et al., 2018). In agreement with these findings, our results showed that EP alleviated the abnormal activation of JAK2 and STAT3 induced by MCAO, while CA1 pretreatment partially diminished the impact of EP. CA1 pretreatment could aggravate the neurological deficits, enlarge the infarct volume, exacerbate neuronal damage, worsen the Th17/Treg imbalance, and intensify the inflammatory response, indicating that EP attenuates cerebral ischemia‐reperfusion injury by inhibiting the JAK2/STAT3 signaling pathway. A graphic representation is shown in Figure 7.

A limitation of the present study was that, similarly to previous research, only young male rats were subjected to the study. Taking female and aged rats into consideration may provide a more comprehensive understanding of the effect of EP because cerebrovascular diseases tend to occur in middle‐aged and older adults. We will consider this aspect in future research. Moreover, further investigations are needed to explore the functional connection of even more molecular pathways and immune factors in the underlying mechanisms of EP.

The focus of our study was to explore the neuroprotective effects of EP. The results support the hypothesis that EP contributes to alleviating MCAO‐induced neuronal apoptosis, Th17/Treg imbalance, and inflammation via inhibiting the JAK2/STAT3 signaling pathway, indicating its therapeutic potential in ischemic stroke.

## AUTHOR CONTRIBUTIONS

Yuan Shan and Le Wang designed the study. Yuan Shan, Le Wang, Jingying Sun, and Sha Chang performed the study. Yuan Shan and Wei Di prepared the manuscript. All the authors have read and approved the submission.

## CONFLICT OF INTEREST STATEMENT

All authors declare no conflict of interest

### PEER REVIEW

The peer review history for this article is available at https://publons.com/publon/10.1002/brb3.3030.

## Data Availability

The data that support the findings of this study are available from the corresponding author upon reasonable request.
